# Real-Time Quaking-Induced Conversion Detection of Bovine Spongiform Encephalopathy Prions in a Subclinical Steer

**DOI:** 10.3389/fvets.2017.00242

**Published:** 2018-01-19

**Authors:** Soyoun Hwang, M. Heather West Greenlee, Anne Balkema-Buschmann, Martin H. Groschup, Eric M. Nicholson, Justin J. Greenlee

**Affiliations:** ^1^United States Department of Agriculture, Agricultural Research Service, National Animal Disease Center, Virus and Prion Research Unit, Ames, IA, United States; ^2^Department of Biomedical Sciences, Iowa State University College of Veterinary Medicine, Ames, IA, United States; ^3^Institute of Novel and Emerging Infectious Diseases, Friedrich-Loeffler-Institut, Federal Research Institute for Animal Health, Greifswald, Germany

**Keywords:** bovine spongiform encephalopathy, diagnosis, prion diseases, prion protein, protein misfolding, real-time quaking-induced conversion, transmissible spongiform encephalopathy

## Abstract

Bovine spongiform encephalopathy (BSE) belongs to a group of fatal prion diseases that result from the misfolding of the cellular prion protein (PrP^C^) into a pathogenic form (PrP^Sc^) that accumulates in the brain. *In vitro* assays such as serial protein misfolding amplification and real-time quaking-induced conversion (RT-QuIC) allow assessment of the conversion of PrP^C^ to PrP^Sc^. RT-QuIC can be used for the detection of prions in a variety of biological tissues from humans and animals. However, there is no such comparison of RT-QuIC data between BSE positive and presymptomatic cattle. Further, the current study assesses prion distribution in multiple brain regions of clinically ill or subclinical animals. Here, we compare RT-QuIC reactions seeded with brain samples collected from experimentally inoculated cattle that were clinically ill or subclinically affected with BSE. The results demonstrate RT-QuIC seeding in various brain regions of an animal with subclinical BSE despite being determined negative by immunohistochemistry. Bioassay of the subclinical animal and RT-QuIC of brainstem from inoculated knockout (*PRNP*^−^*^**/**^*^−^) cattle were used to confirm infectivity in the subclinical animal and determine that RT-QuIC reactions were not the result of residual inoculum, respectively. These results confirm that RT-QuIC is a highly sensitive prion detection assay that can detect prions in a steer prior to the onset of clinical signs of BSE.

## Introduction

Bovine spongiform encephalopathy (BSE) belongs to a group of fatal neurologic diseases that result from the misfolding of the cellular prion protein (PrP^C^) into a pathogenic form (PrP^Sc^) in the brain (1–3). Collectively, these diseases are referred to as prion diseases or transmissible spongiform encephalopathies (TSEs). In addition to BSE, the prion diseases include scrapie in sheep, chronic wasting disease (CWD) in cervids, and Creutzfeldt-Jakob disease (CJD), fatal familial insomnia, Gerstmann-Sträussler-Scheinker syndrome, and kuru in humans. Classical BSE is a feedborne disease in cattle and transmits to humans as variant CJD (4, 5).

Currently approved BSE diagnostic tests are based on the direct detection of PrP^Sc^ by immunoblot, enzyme immunoassay (EIA or ELISA), or immunohistochemistry (IHC) (6, 7). New prion detection tools relying on the *in vitro* amplification of PrP^Sc^ have been developed and include protein misfolding cyclic amplification (PMCA) (8–10) and the real-time quaking-induced conversion (RT-QuIC) assay (11–18). Both of these approaches can amplify very low levels of PrP^Sc^ to levels that are readily detectable. PMCA is a very useful methodology for sensitive detection of prions in various samples of different species, but it requires an animal derived substrate (usually brain homogenate) and is time consuming because final analysis requires Western blotting. Unlike PMCA, RT-QuIC is a relatively simple technique that makes use of recombinant prion protein as a substrate. This technique monitors the fibril formation in real-time *via* binding of the fluorescent marker, thioflavin T (ThT), to the resulting amyloid fibrils. ThT fluorescence increases as the amount of amyloid fibril accumulates through conversion of monomeric PrP^C^ to an amyloid conformation. The RT-QuIC assay has been shown to detect low levels of TSE infectivity and to detect human and animal prions in various tissues (11–18).

Even after experimental inoculation by the intracranial route, there is a period of time during incubation where PrP^Sc^ is undetectable in brain samples when assayed by traditional diagnostic methods for prion diseases (19). Amplification based assays, such as RT-QuIC represent an opportunity for evaluation of PrP^Sc^ distribution in situations where the level is anticipated to be low, such as during the preclinical incubation period. Currently, there is a lack of RT-QuIC data on detection of prion diseases in subclinical cattle. Here, we applied RT-QuIC to assess relative levels and distribution of PrP^Sc^ among cattle in various groups: clinically affected with BSE; BSE inoculated, but without clinical signs of disease (subclinical); inoculated knockouts (*PRNP*^−^*^/^*^−^) that did not develop clinical disease after long incubation periods, and sham-inoculated controls.

## Materials and Methods

### Ethics Statement

The laboratory and animal experiments were conducted in Biosafety Level 2 spaces that were inspected and approved for importing prion agents by the US Department of Agriculture, Animal and Plant Health Inspection Service, Veterinary Services. The studies were done in accordance with the Guide for the Care and Use of Laboratory Animals (Institute of Laboratory Animal Resources, National Academy of Sciences, Washington, DC, USA) and the Guide for the Care and Use of Agricultural Animals in Research and Teaching (Federation of Animal Science Societies, Champaign, IL, USA). The protocols were approved by the Institutional Animal Care and Use Committee at the National Animal Disease Center (protocol numbers: 3636 and 3985), which included a biosafety review. Cattle were monitored twice daily for the appearance of clinical signs suggestive of prion disease such as aggression, behavior changes, decreased feed intake, loss of body condition, ataxia, prolonged recumbency, or inability to rise. All inoculated animals developed the clinical signs specifically described in Table 1 and no animals died prior to the experimental endpoint. To ensure a humane endpoint, animals were euthanized if they demonstrated clinical signs of prolonged anorexia (>24 h) or recumbency (>12 h). Animals exhibiting behavioral or postural changes consistent with TSE were examined by a veterinarian. Upon veterinary confirmation of unequivocal clinical signs of TSE, affected cattle were killed with an intravenous injection of pentobarbital sodium according to the manufacturer’s directions and necropsied. Any other concurrent diseases were treated under the direction of a veterinarian.

**Table 1:** Inoculum and clinical history for experimental cattle.

Animal number	Inoculum	Source of inoculum	Days postinoculation	Age at death (month)	Signs	Clinical description
1	Classical BSE	US 2003 (WA)	939	86	−	No clinical signs
2	Classical BSE	US 2003 (WA)	687	77	+	Sawhorse stance, increased startle response, goose-stepping gate
3	Classical BSE	US 2003 (WA)	639	76	+	Sawhorse stance, irritable when encouraged to walk, fell down while walking
4	TME	Second passage	689	31.8	−	No clinical signs
5	TME	Second passage	1,433	54.3	−	No clinical signs
6	Sham	NBH	1,667	80.7	−	No clinical signs

*NBH, normal brain homogenate*.

### Brain Samples from Experimentally Inoculated Cattle

Cattle (*n* = 12) were intracranially inoculated with 1 ml of a 10% (wt./vol.) brain homogenate from a cow diagnosed with classical BSE in the US in 2003 as previously described (20). The *PRNP* gene of all inoculated animals was sequenced. The predicted amino acid sequences from inoculated cattle were similar to each other and consistent with previously reported cattle sequences (21). Brain samples from various regions including brainstem at the level of the obex, cerebellum, midbrain, thalamus, and cerebrum were obtained from cattle in the following experimental groups: (1) wild-type cattle intracranially sham-inoculated with normal brain homogenate (negative controls), (2) wild-type cattle intracranially inoculated with classical BSE (22), or (3) knockout cattle (*PRNP*^−/−^) intracranially inoculated with the transmissible mink encephalopathy (TME) agent. Homogenates were prepared from brainstem sections of TSE-free cattle or cattle with clinical signs of TME (23–25) or classical BSE by homogenization with 1.0 mm silica beads in 1× Dulbecco’s PBS pH 7.4 to produce a 10% w/v homogenate. Samples were centrifuged briefly at 1,000 *g* for 2 min and supernatants were separated and stored at −80°C in aliquots. The sham-inoculated negative control animal was housed separately from the other cattle in this study. All animal donor information including TSE isolate inoculated, source of inoculum, incubation periods, and description of clinical signs are shown in Table 1.

### Microscopic Examination and IHC

After fixation in 10% neutral buffered formalin, tissues were embedded in paraffin and processed by routine methods for microscopic examination after staining by hematoxylin and eosin or by immunohistochemical methods as described previously (26). Sections of cerebrum, thalamus, midbrain, and brainstem at the level of the obex were assessed for spongiform change and PrP^Sc^ accumulation. After deparaffinization and rehydration, sections (4 µm on charged slides) were autoclaved for 20 min in antigen retrieval solution (DAKO Target Retrieval Solution, DAKO Crop., Carpinteria, CA, USA), and stained with an indirect, alkaline phosphatase-labeled secondary antibody (ultraview Universal Alkaline Phosphatase Red Detection Kit, Ventana Medical Systems, Inc., Tucson, AZ, USA). The primary antibody was mAb F99/97.6.1 (27) used at 10 µg/ml for a 32 min incubation at 37°C. Slides were counterstained with Gill’s hematoxylin and bluing agent (Ventana Medical Systems, Inc., Tucson, AZ, USA) prior to coverslipping.

### Western Blotting of Cattle Brain Homogenates

All brain tissues including one sham-inoculated negative control, two TME-infected *PRNP* knock out cattle, three BSE-infected cattle: two with clinical signs (animal No. 2 and No. 3) and one without clinical signs (animal No. 1) were run on the Western blot (WB). Each sample was digested with 50 µg of proteinase K (PK) for 1 h at 42°C and separated by SDS-PAGE on 12% polyacrylamide minigels (Invitrogen) and then transferred onto polyvinylidene difluoride membranes (Millipore, Billerica, MA, USA) for 45 min at 30 V. Bovine serum albumin (3%) in Tris-buffered saline was used to block the membranes for 1 h and then membrane was incubated with mouse anti-PrP monoclonal antibody 6H4 as the primary antibody at 1:10,000 dilution (0.1 µg/ml) for another hour. Then, a biotinylated sheep antimouse secondary antibody at 0.05 µg/ml and a streptavidin–horseradish peroxidase conjugate were used in conjunction with a chemiluminescent detection system (Pierce ECL plus, Thermo Fisher) and visualized on an imaging system capable of detecting luminescence.

### EIA of Brain Homogenates from Cattle

Enzyme immunoassay was performed to determine the relative amount of misfolded protein in brain homogenates from all animals including one sham-inoculated, two TME-infected *PRNP* knock out cattle, three BSE-infected cattle: two with clinical signs (animal No. 2 and No. 3) and one without clinical signs (animal No. 1) using an IDEXX HerdChek BSE EIA kit without treatment of PK. The cutoff value was determined by the negative control sample provided by the manufacturer and the optical density value was around 0.08 ± 0.005. Samples were considered positive if the optical density value was over 0.15.

### PK Sensitivity of Brain Homogenates from Cattle

Brain homogenates (10% w/v) prepared in PBS were digested or treated with different concentrations of PK (0–0.5 mg/ml; VWR, Visalia, CA, USA) for 1 h at 37°C. 1× pefabloc proteinase inhibitor (VWR, Visalia, CA, USA) was added to terminate the reaction. The relative amount of misfolded protein was measured by IDEXX HerdChek BSE Kit (see above). Each sample was performed in triplicate.

### Recombinant Prion Protein Production and Purification

*Escherichia coli* [BL21(λDE3)] transformed with the pET28a vector harboring the wild-type bovine PrP gene (amino acids 25–241; GenBank accession number: DQ875147.1) was grown, and the bovine prion protein was expressed and purified as described by Vrentas et al. (28). The concentration of purified pooled protein was measured by UV spectroscopy and calculated from the absorbance at 280 nm using an extinction coefficient of 63,495 M/cm as calculated for wild-type protein.

### RT-QuIC Protocol

Real-time quaking-induced conversion reactions were performed as previously described (29). The RT-QuIC reaction buffer was composed of 10 mM phosphate buffer (pH 7.4), 300 mM NaCl, 0.1 mg/ml recombinant bovine prion protein, 10 µM ThT, 1 mM ethylenediaminetetraacetic acid tetrasodium salt, and 0.001% SDS. A volume of 98 µl of the RT-QuIC reaction buffer was loaded into each well of a black 96-well plate with a clear bottom (Nunc, Thermo Fisher Scientific) and 2 µL of brain homogenate dilution was added to test for seeding activity. Brain homogenate (10% w/v) was prepared as previously described (11) and stored at −80°C.

The plate was sealed and incubated in a BMG FLUOstar Omega plate reader at 42°C for 100 h with cycles of 15 min shaking (700 rpm double orbital pattern) and 15 min rest. ThT fluorescence measurements (460 nm excitation and 480 nm emission) were taken every 15 min, with the gain set at 1,400. All reactions for each sample were performed in eight replicates (quadruplicates in two independent RT-QuIC assays). ThT fluorescence data are displayed as the average ThT fluorescence of four technical replicates for each time point and, to be considered positive, the ThT fluorescence of at least two replicate reactions must be positive. The predefined positive threshold was calculated as 10 SDs above the mean fluorescence of normal cattle brain homogenates. Previously described criteria were applied for classification of positive samples of RT-QuIC (18, 30, 31).

### Mouse Bioassay

To further characterize the potential infectivity of brain material from animal No. 1 and allow for comparison with RT-QuIC results, bioassays were conducted in TgBovXV (32) mice under isoflurane anesthesia as previously described (33). Inocula were derived from the brainstem of subclinical bovine No. 1 (10% w/v in PBS) or the original classical BSE inoculum (1% w/v in PBS) (34). Each mouse was inoculated with 20 µl intracranially. Mice were monitored daily for the development of clinical signs by animal care staff. When signs suggestive of prion disease such as ataxia, listing or rolling gate, pelvic limb paresis, lethargy, or poor grooming with urine stained fur were recognizable by observation, animals were humanely euthanized and their brains prepared for analysis by EIA as described above. The attack rate was defined as the percentage of mice from an experimental group that had a positive EIA result. Incubation periods are expressed as the mean days postinoculation (days PI) for mice with positive EIA results. All animals that died due to intercurrent disease or without the development of clinical signs 2 SDs or less prior to the average incubation time were included in the calculation of attack rate. Using these criteria to calculate attack rate, 18 mice were used for the classical BSE group and 16 mice were included in the No. 1 inoculation group.

## Results

### Incubation Periods and Clinical Signs

The incubation period for classical BSE in cattle is reported here as the time from inoculation to the time when unequivocal signs of clinical disease were present. Clinical signs of disease included abnormalities in gait or stance, moderate to severe ataxia, and hyperreaction to stimuli such as noise or movement. The average incubation period was approximately 22 months (22). One animal (No. 1) was euthanized at the termination of the study at 31 months without developing clinical signs (Table 1) despite receiving the same inoculum by intracranial inoculation without any noted deviations in procedure. Tissues from this animal were subjected to additional studies in an effort to explain the failure to develop clinical signs.

### Microscopic Examination and IHC

**Figure 1:**
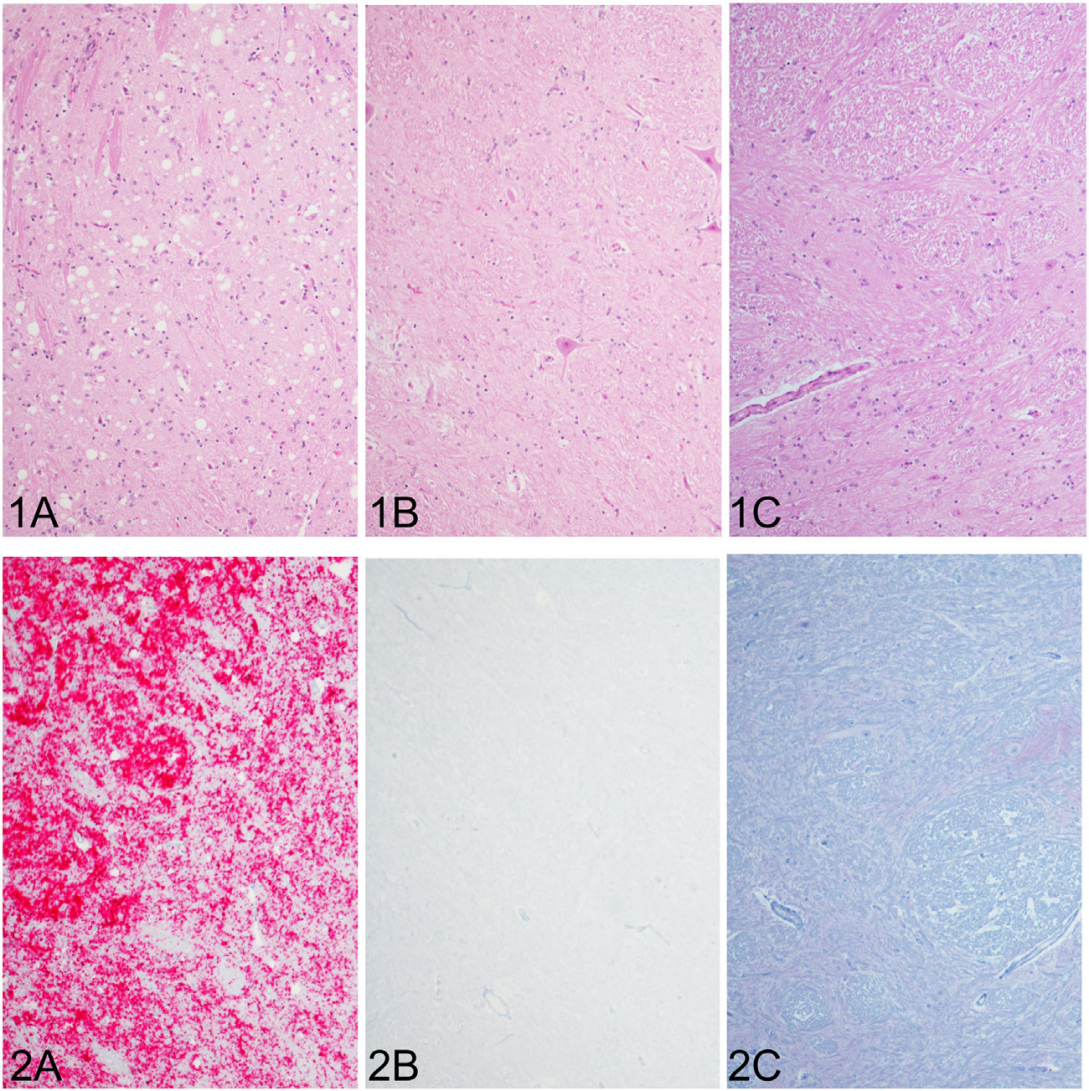
Comparison of spongiform change and PrP^Sc^ accumulation in the brainstem at the level of the obex (all images of spinal tract and nucleus of the trigeminal nerve). Spongiform change is severe in the sections of brain steer No. 2 that had clinical signs of bovine spongiform encephalopathy (BSE) **(1A)** and corresponds with intense immunoreactivity for PrP^Sc^ [red chromogen; **(2A)**]. Neither spongiform change nor PrP^Sc^ immunostaining was observed in brain sections of a steer No. 1 with subclinical BSE **(1B,2B)**, which appear similar to a sham-inoculated, negative control steer **(1C,2C)**. Original magnification of all images is 10×. Immunohistochemistry done with mAb F99/97.6.1.

In the cattle clinically affected with BSE, PrP^Sc^ immunoreactivity was detected in all brain regions examined and corresponded with spongiform change. Spongiform change was most severe in the brainstem (nucleus of the solitary tract and nucleus of the spinal tract of the trigeminal nerve (Figure 1A1), caudal midbrain, and hypothalamus. PrP^Sc^ immunolabeling in the steer No. 2 (clinically affected with BSE) included granular, linear, aggregated, intraneuronal, and stellate labeling types. There was moderately strong immunolabeling of the cerebrum (neocortex) with prominent stellate and intraglial labeling and less granular and intraneuronal labeling compared to the thalamus, midbrain) and brainstem (Figure 1A2). There was no evidence of spongiform change or PrP^Sc^ immunoreactivity in any of the other groups represented: inoculated with the BSE agent but prior to the onset of clinical signs (steer No. 1, Figures 1B1,B2); knockout cattle (*PRNP*^−/−^) inoculated with the TME agent, or a sham-inoculated control animal (Figures 1C1,C2).

### Western Blotting in Brain Samples from Clinical, Subclinical, Knockout, and Sham-Inoculated Cattle

**Figure 2:**
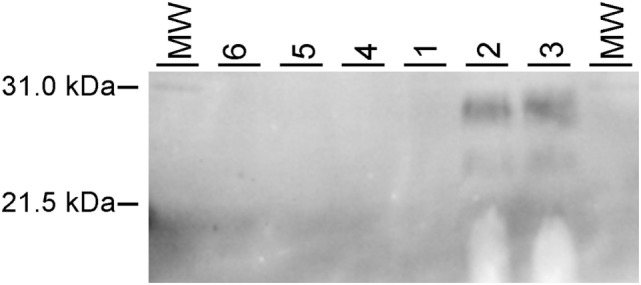
Western blot analysis of relative amounts of PrP^Sc^ in brain homogenates from bovine spongiform encephalopathy (BSE)-infected cattle. Each lane represents proteinase K (PK)-digested brain homogenate probed with an anti-PrP monoclonal antibody 6H4. MW, molecular weight marker. Lanes are labeled with animal number: 6—sham-inoculated, 5 and 4—TME-inoculated knockout cattle, 1—steer with subclinical BSE, and 2 and 3—cattle with clinical evidence of BSE.

Samples of brainstem at the level of the obex were collected from representative cattle. WBs were done using the monoclonal antibody 6H4 to demonstrate PrP^Sc^. WB results are shown in Figure 2. WB confirmed evidence of PrP^Sc^ in cattle with clinical signs (Nos. 2 and 3), but failed to demonstrate PrP^Sc^ in cattle necropsied prior to the onset of clinical signs (No. 1), knockout cattle inoculated with ME (Nos. 4 and 5), or a sham-inoculated steer (No. 6).

### Quantitation of PrP^Sc^ by EIA in Brain Samples from Clinical, Subclinical, Knockout, and Sham-Inoculated Cattle

Enzyme immunoassay was initially performed to on brainstem samples from all BSE-inoculated cattle to determine the relative amounts of misfolded prion protein present. Similar to IHC and WB, no misfolded prion protein was detected in the brainstem of steer No. 1, whereas high levels were detected in the brainstem of cattle with clinical signs (steers No. 2 and No. 3; see Table 2).

**Table 2:** IDEXX HerdChek enzyme immunoassay results of various brain regions from experimental animals.

Animal no.	OD for brainstem	OD for cerebrum	OD for cerebellum	OD for midbrain	OD for thalamus
1 (Subclinical)	0.065	0.115	0.116	2.53	3.26
2 (Clinical)	3.3	1.6	1.3	4.0	0.28
3 (Clinical)	2.5	0.19	0.19	2.2	4.0
4 (KO)	0.08	0.08	0.07	0.075	0.077
5 (KO)	0.075	0.073	0.073	0.077	0.073
6 (Sham-inoculated)	0.08	0.068	0.062	0.073	0.069

*Positive cutoff value defined as 0.15. All positive samples are highlighted in blue*.

Steer No. 1 was inoculated with brain homogenate of known infectivity, but brainstem samples tested negative by EIA. Therefore, EIA was performed on additional brain regions including cerebrum, cerebellum, midbrain, and thalamus. Samples from cerebrum and cerebellum also showed low OD similar to the negative control. Interestingly, relatively high levels of PrP^Sc^ were detected in the midbrain and thalamus regions (Table 2). When the same brain regions were tested from cattle with clinical signs, they were strongly positive by EIA. None of the brain regions sampled from the sham-inoculated control or knockout animals inoculated with TME were positive by EIA.

### RT-QuIC Seeding Activity in Brainstem Samples from Clinical, Subclinical, and Sham-Inoculated Control Cattle

**Figure 3:**
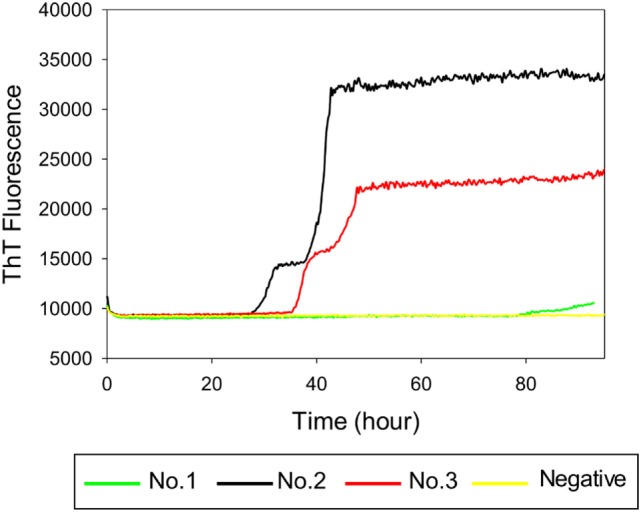
Real-time quaking-induced conversion (RT-QuIC) detection of classical bovine spongiform encephalopathy prion seeding activity using recombinant bovine prion protein. RT-QuIC reaction mixtures were seeded with 10^−4^ dilutions of brain homogenate from classical BSE-affected cattle (animal No. 2 and No. 3), steer No. 1, or a negative control animal. A final SDS concentration of 0.001% in combination with 300 mM NaCl was used with the substrate. Data are presented as mean thioflavin T (ThT) fluorescence of eight repeated reactions. The positive threshold was calculated as ~10,000 relative fluorescence units of brain homogenate from normal cattle.

To investigate whether RT-QuIC can detect prion seeding activity from cattle prior to the onset of clinical signs of classical BSE, we compared brainstem homogenates from each of the experimental groups using the RT-QuIC assay with full length bovine rPrP [a.a. 25–241] substrate and the reaction conditions (0.1 mg/ml rPrP final concentration, 300 mM NaCl, and 0.001% SDS) as previously used (29). An increase in ThT fluorescence was observed within 40 h incubation in each quadruplicate reaction seeded with 10^−4^ dilution of 10% w/v classical BSE brain homogenate including animal No. 2 and No. 3 (Figure 3). However, fluorescence was not produced in assays seeded with either brainstem of steer No. 1 or negative control animals indicating extremely low or absent prion levels in the brainstem of those animals.

### RT-QuIC Seeding Activity in Various Brain Regions of Cattle with Subclinical BSE

**Figure 4:**
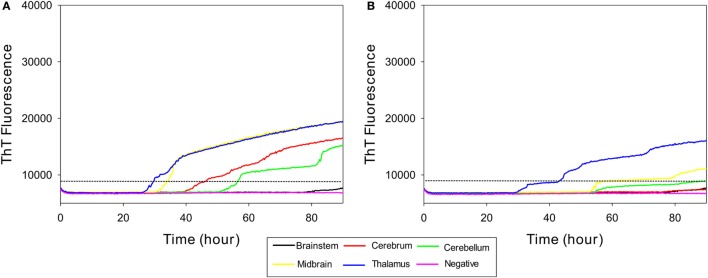
Real-time quaking-induced conversion (RT-QuIC) detection of prion seeding activity from each section of brain of a subclinical animal. Bovine recombinant prion protein was used as a substrate for RT-QuIC reactions. RT-QuIC reaction mixtures were seeded with 10^−2^ **(A)** and 10^−4^ **(B)** dilutions of brain tissues including brainstem, cerebrum, cerebellum, midbrain, and thalamus. A final SDS concentration of 0.001% in combination with 300 mM NaCl was used with the substrate. Data are presented as mean thioflavin T (ThT) fluorescence of quadruplicate reactions. The positive threshold was calculated as ~10,000 relative fluorescence units of brain homogenate from normal cattle.

In order to determine whether RT-QuIC can detect PrP^Sc^ in other brain regions of subclinical cattle, samples from multiple brain regions of a steer No. 1 were assessed. We compared 5 brain regions including brainstem at the level of the obex, cerebrum, cerebellum, midbrain, and thalamus to a negative control sample. As can be seen in Figure 4A, all reactions seeded with the four brain regions (except brainstem) gave strong positive responses within 60 h for the 10^−2^ brain dilutions. The reaction seeded with thalamus had the shortest lag time about 25 h. Reactions seeded with cerebrum and cerebellum also produced an increase in ThT fluorescence (Figure 4A) despite having OD values similar to the negative control of the EIA kit confirming that RT-QuIC is more sensitive than the EIA assay for prion detection. For the 10^−4^ brain dilution (see Figure 4B), a thalamus sample resulted in ThT fluorescence while assays with other brain regions showed weak or negative ThT responses.

### RT-QuIC Seeding Activity of Samples from Cattle with Clinical Signs of Classical BSE

**Figure 5:**
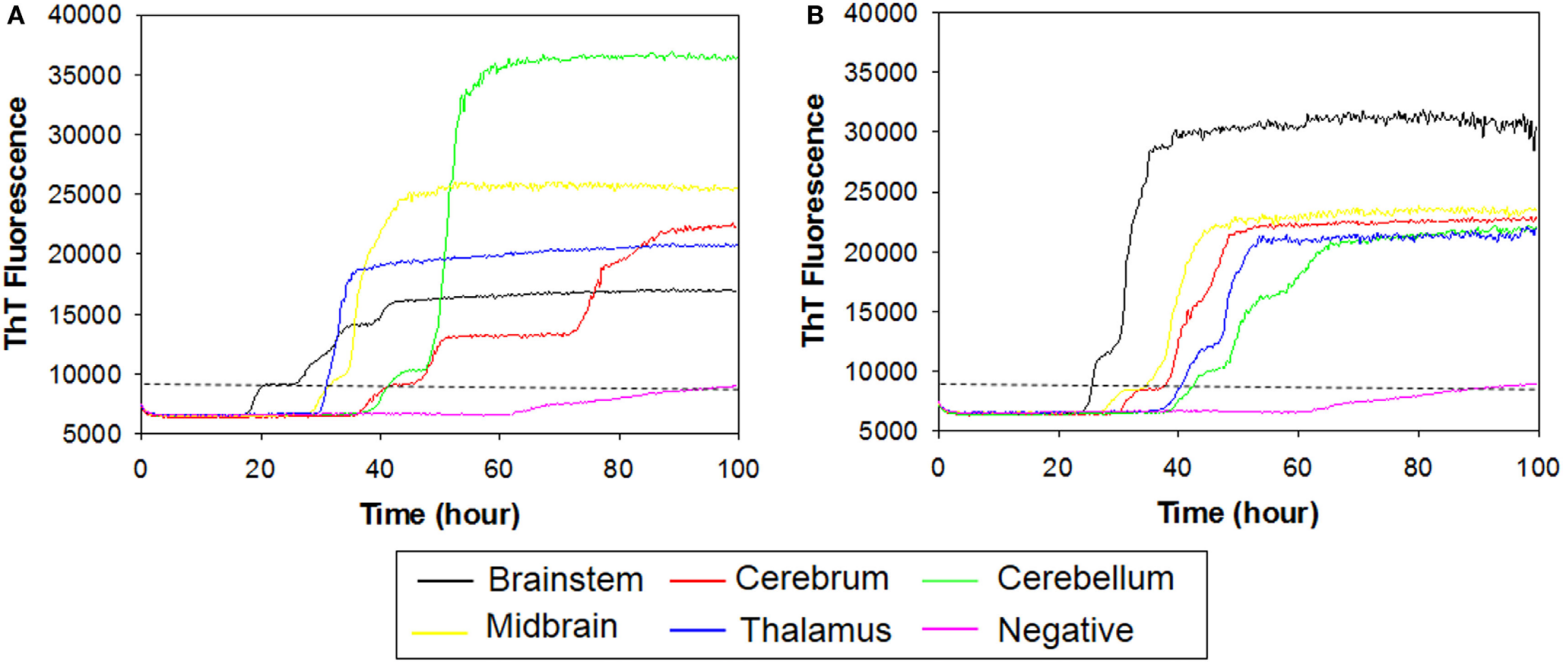
Real-time quaking-induced conversion (RT-QuIC) detection of prion seeding activity from each brain region of positive animals including animal No. 2 and No. 3. Recombinant bovine prion protein was used as a substrate for all RT-QuIC reactions. RT-QuIC reaction mixtures were seeded with 10^−2^ dilutions of brain tissues including brainstem, cerebrum, cerebellum, midbrain, and thalamus of either animal No. 2 **(A)** or No. 3 **(B)**. A final SDS concentration of 0.001% in combination with 300 mM NaCl was used with the substrate. Data are presented as mean thioflavin T (ThT) fluorescence of eight repetitions. The positive threshold was calculated as ~10,000 relative fluorescence units of brain homogenate from normal cattle.

To further compare RT-QuIC reactions of steer No. 1 with those of cattle clinically ill with BSE, we tested brain samples from multiple regions. Table 1 summarizes characteristics of two animals including incubation period, inoculum source, and clinical signs. RT-QuIC detection of prion seeding activity from each brain region from cattle with clinical signs of BSE is demonstrated in Figure 5. RT-QuIC reaction mixtures were seeded with 10^−2^ dilutions of brain tissues including brainstem at the level of the obex, cerebrum, cerebellum, midbrain, and thalamus of either animal No. 2 (Figure 5A) or No. 3 (Figure 5B). As can be seen in Figure 5, reactions seeded with brainstem at the level of the obex from animals with clinical signs of BSE had a relatively shorter lag time than other reactions seeded with any other brain sections. Reactions seeded with samples from either the thalamus or the midbrain had slightly longer lag times than reactions seeded with samples from the brainstem. Reactions seeded with samples from the cerebrum and the cerebellum also produced ThT fluorescence with longer lag time than midbrain or thalamus seeded reactions. All reactions seeded with negative brain failed to demonstrate increases in ThT fluorescence.

### PK Sensitivity PrP^Sc^ from the Brains of Cattle Inoculated with Classical BSE

**Figure 6:**
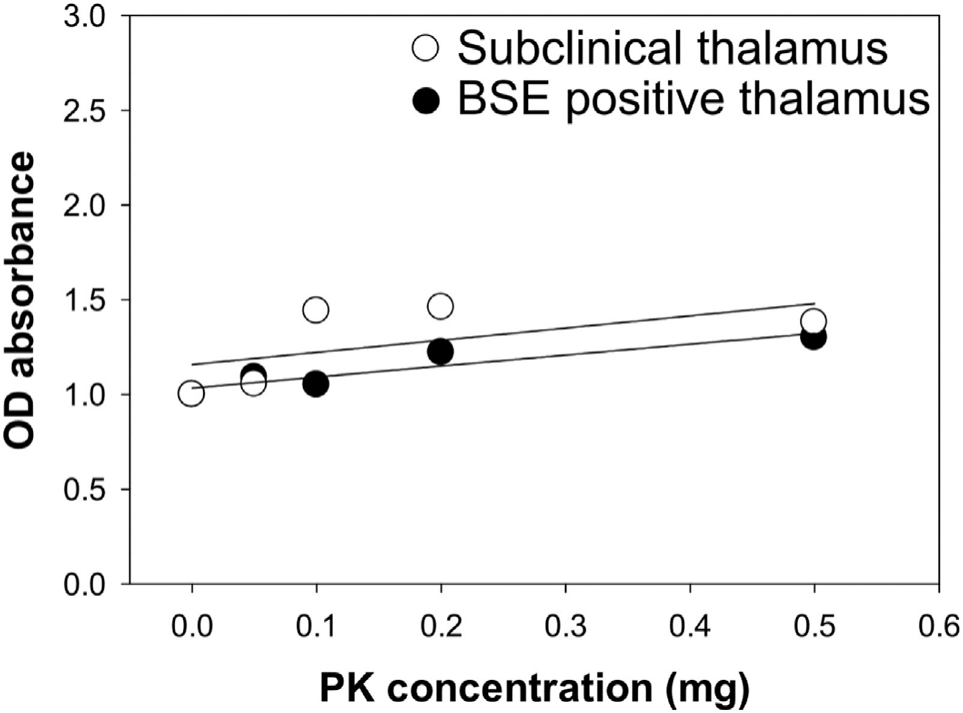
Assessment of proteinase K (PK) sensitivity of PrP^Sc^ from the brains of bovine spongiform encephalopathy (BSE)-inoculated cattle. Brain samples from a subclinical animal (open circle) and animal with clinical signs of BSE (closed circle) were treated with increasing concentrations of PK.

Samples from steer No. 1 were negative by IHC and WB, but positive in midbrain and thalamus using an EIA method that binds misfolded proteins rather than testing for protease resistance. Therefore, we wanted to test PrP^Sc^ from the midbrain and thalamus of steer No. 1 for potential PK sensitivity. PK sensitivity testing was performed by measuring the absorbance of bound, misfolded protein before and after treatment with PK using EIA as described above. Brain homogenates of thalamus region from steers No. 1 and No. 2 were prepared in PBS and treated with various concentrations of PK (0–0.5 mg/ml). The results demonstrated that stable absorbance values after treatment with increasing amounts of PK in all samples (Figure 6).

### Comparison of RT-QuIC Seeding Activity of Samples from Cattle with Subclinical BSE to Samples from TME-Inoculated PRNP^−/−^ Knockout Cattle

**Figure 7:**
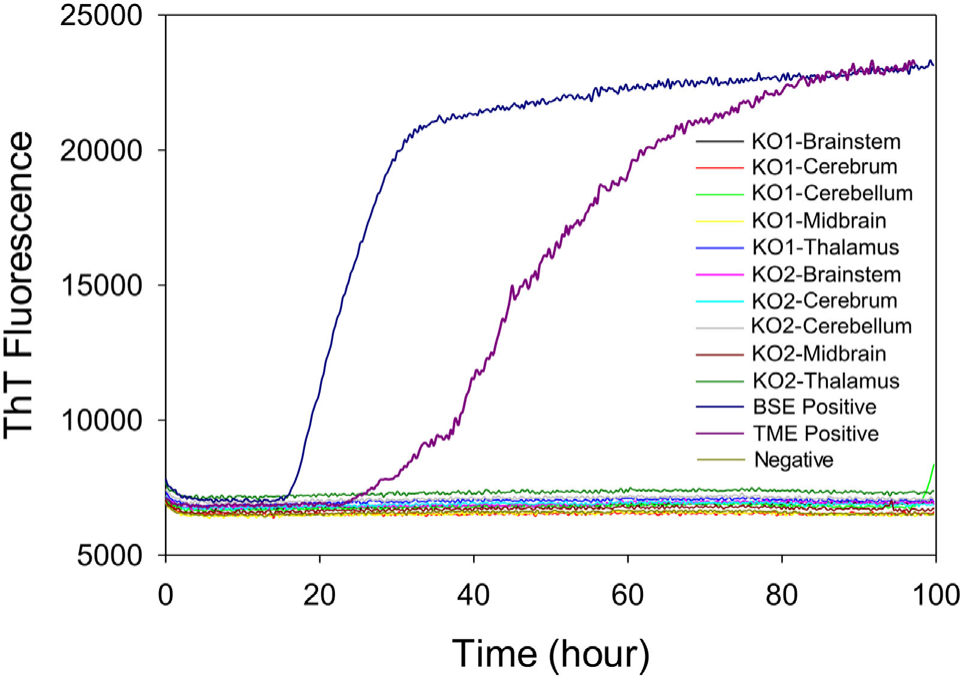
Comparison of real-time quaking-induced conversion reactions using different brain regions of samples from prion knockout (*PRNP*^−^*^/^*^−^) animals or negative or positive controls. A final SDS concentration of 0.001% in combination with 300 mM NaCl was used with the substrate. Data are presented as mean thioflavin T (ThT) fluorescence of quadruplicate reactions. The positive threshold was calculated as ~10,000 relative fluorescence units of brain homogenate from normal cattle.

To test whether the ThT fluorescence response of samples from the midbrain and thalamus of steer No. 1 resulted from the residual inoculum, we measured RT-QuIC reactions seeded with brains from knockout (*PRNP*^−/−^) cattle that were intracranially inoculated with TME. We evaluated whether different brain regions of intracranially inoculated knockout animals produced similar RT-QuIC responses to steer No. 1. Prion seeding activity was not detected in any RT-QuIC reactions seeded with brain samples from *PRNP* knockout cattle, whereas assays with brainstem samples from cattle with clinical signs of BSE or TME demonstrate strong ThT fluorescence (Figure 7).

### Mouse Bioassay

**Figure 8:**
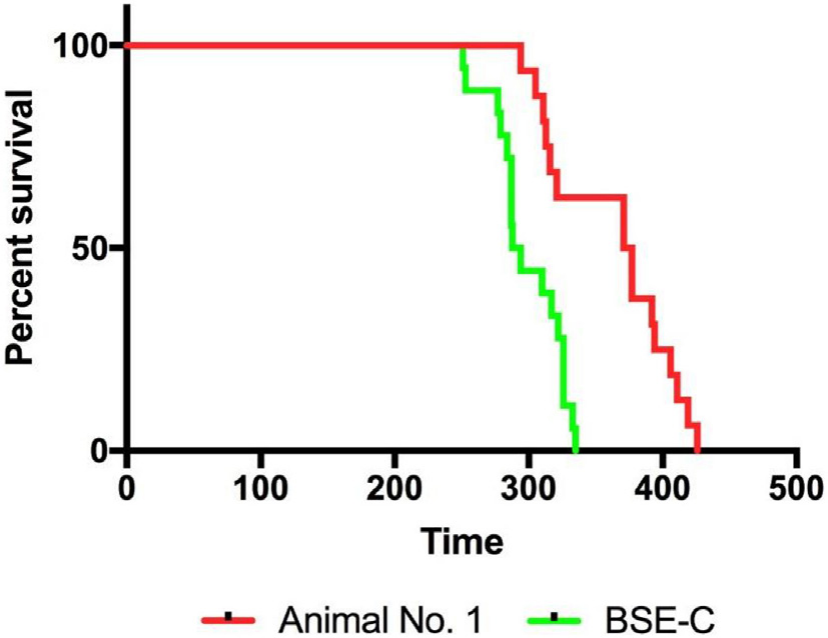
Infectivity was present in the brainstem of steer No. 1 despite testing negative by immunohistochemistry, Western blot, and real-time quaking-induced conversion. TgBov XV mice inoculated with a 1% homogenate of the original classical bovine spongiform encephalopathy (BSE) inoculum had 100% attack rate and a mean incubation period of 299 days (green line). Mice inoculated with a 10% homogenate from the brainstem of the subclinical steer No. 1 also had a 100% attack rate but with a prolonged mean incubation period of 363 days (red line). The attack rate was defined as the percentage of mice that had a positive enzyme immunoassay (EIA) result from an experimental group that includes all mice that died within 2 SDs of the mean incubation period. Incubation periods are expressed as the mean days postinoculation (days PI) for mice with positive EIA results.

To allow for comparison of RT-QuIC results with that of bioassay, we inoculated mice with the original classical BSE inoculum or brain homogenate from subclinical steer No. 1. Mice inoculated with 1% classical BSE inoculum (18/18; 100%) developed clinical signs of difficulty moving and poor coordination and were euthanized with a mean incubation period of 299 days. Mice inoculated with 10% brainstem homogenate from steer #1 developed clinical similar signs (16/16; 100%), but with a longer mean incubation period of 363 days despite receiving a more concentrated brain homogenate (Figure 8).

## Discussion and Conclusion

With this study, we provide proof of concept that RT-QuIC is a highly useful method for the detection of BSE prions in the brains of cattle with subclinical BSE. In the current study, an animal inoculated with classical BSE (No. 1) did not develop clinical illness and tested negative by standard diagnostics performed on brainstem despite a prolonged observation period of 31 months. When brainstem tested negative by EIA, we tested additional brain regions by EIA and RT-QuIC and compared the results to those of animals clinically ill with BSE in an effort to explain the failure of this animal to develop clinical signs. Prion distribution differences between subclinical and clinical animals led us to perform additional RT-QuIC reactions in *PRNP* knockout animals to ensure our RT-QuIC results were not due to the presence of residual TSE inoculum. Prions were not detected in brain samples from *PRNP* knockout animals by WB, EIA, IHC, or RT-QuIC.

In the current study, RT-QuIC detected lower levels of prions than traditional diagnostic tools. IHC and WB failed to identify any PrP^Sc^ in the subclinical animal (No. 1), while testing multiple brain regions by EIA and RT-QuIC allowed us to detect misfolded protein in midbrain and thalamus. Further, we were able to detect seeding activity in brain regions including cerebrum and cerebellum that were negative by EIA. This result is consistent with a recent study that demonstrated that RT-QuIC is more sensitive for the detection of PrP^CWD^ during early infection than tyramide signal amplification-IHC (35).

Real-time quaking-induced conversion has been successfully used for sensitive and specific detection of prion diseases for humans and animals (11–13, 15–17, 36). We previously reported different types of BSE prions can be detected and differentiated using RT-QuIC with recombinant bovine prion protein (29), and the previous work of others evaluated detection and discrimination of classical, H-, and L-BSE prions by full-length chimeric hamster-sheep prion proteins, N-terminally truncated hamster prion protein [90–231] (37), full-length sheep protein, and full-length bank vole protein (a.a. 23–230). There has been significant effort to detect prions in presymptomatic diagnostic samples including CSF, blood, urine, saliva, and nasal brushings by RT-QuIC [7–16]. CWD prions were detected in urine collected from presymptomatic deer and in fecal extracts by using RT-QuIC (38) suggesting potential applications in CWD surveillance and control.

In the present study, there were differences in prion distribution between the brains of subclinical and clinically affected cattle after intracranial BSE inoculation. In the subclinically affected steer (No. 1), the highest levels of prions accumulated in the thalamus and midbrain, while no prions were detected in the brainstem. Most likely this reflects prion accumulation in the location that the inoculum was deposited without further spread into other brain regions. A study comparing preclinical cattle infected naturally with BSE to clinically affected cattle either naturally or experimentally infected with BSE by the oral route found the most abundant PrP^Sc^ in the brainstem area (39), which is consistent with ascension to the brain from the gut by sympathetic and parasympathetic projections (40). In our experiment, abundant prions were observed in the brainstem of cattle with clinical signs of BSE, which is similar to the amount in their thalamus or midbrain regions. Interestingly, prions in the brainstem of cattle with clinical evidence of BSE seeded the RT-QuIC reactions faster than any other brain region despite the brainstem area having lower EIA OD values (Table 2) in comparison to other brain regions. This suggests that higher concentrations of prions do not necessarily seed the reaction faster. Perhaps prions of the brainstem exist in a preferred conformation for better conversion despite being present in lower concentrations.

Bioassay of brainstem from steer No. 1 confirmed infectivity and RT-QuIC results despite being negative by IHC, WB, and EIA. Bioassay was performed with a 10% homogenate as compared to 1% for the original classical BSE inoculum, yet the average incubation period longer confirming lower infectivity in the brainstem of the subclinical steer. We previously reported that the retinas of animals inoculated with the agent of classical BSE become substantially thinner than both their baseline (preinoculation) thickness and the thickness of sham-inoculated control animals (22). This decrease in retinal thickness preceded the onset of clinical signs by an average of 10 months. It is interesting to note that in the previous study steer No. 1 had a substantially decreased retinal thickness value at 12 months postinoculation and would have been classified as “positive” by these previously reported criteria (22).

After being observed for nearly 9 months longer than other cattle in the same experimental group, a steer intracranially inoculated with classical BSE was euthanized without demonstrating clinical signs suggestive of prion disease. WB and IHC failed to demonstrate PrP^Sc^ in any region of the brain. In an effort to investigate the reason that this animal failed to develop clinical signs, further studies were conducted using RT-QuIC, which demonstrated high levels of prions in the thalamus and midbrain. In summary, we demonstrate that RT-QuIC can be useful to detect prions in cattle with subclinical BSE and that BSE prions can be differently distributed in the brain regions as incubation progresses.

## Ethics Statement

The animal experiments were carried out in accordance with the Guide for the Care and Use of Laboratory Animals (Institute of Laboratory Animal Resources, National Academy of Sciences, Washington, DC, USA) and the Guide for the Care and Use of Agricultural Animals in Research and Teaching (Federation of Animal Science Societies, Champaign, IL, USA). The protocols were approved by the Institutional Animal Care and Use Committee at the National Animal Disease Center (protocol numbers: 3636 and 3985).

## Author Contributions

Conceived and designed the experiments: SH, MG, and JG. Performed the experiments: SH and JG. Analyzed the data: SH, MG, EN, and JG. Contributed reagents/materials/analysis tools: AB-B, MG, and JG. Wrote the article: SH, MG, and JG. All authors read and approved the final manuscript.

## Disclaimer

Mention of trade names or commercial products in this publication is solely for the purpose of providing specific information and does not imply recommendation or endorsement by the US Department of Agriculture. USDA is an equal opportunity provider and employer.

## Conflict of Interest Statement

The authors declare that the research was conducted in the absence of any commercial or financial relationships that could be construed as a potential conflict of interest.
